# Cancer Incidence among Pesticide Applicators Exposed to Permethrin in the Agricultural Health Study

**DOI:** 10.1289/ehp.11318

**Published:** 2008-11-10

**Authors:** Jennifer A. Rusiecki, Rahulkumar Patel, Stella Koutros, Laura Beane-Freeman, Ola Landgren, Matthew R. Bonner, Joseph Coble, Jay Lubin, Aaron Blair, Jane A. Hoppin, Michael C.R. Alavanja

**Affiliations:** 1 Department of Preventive Medicine and Biometrics, Uniformed Services University, Bethesda, Maryland, USA;; 2 Division of Cancer Epidemiology and Genetics, National Cancer Institute, National Institutes of Health, Department of Health and Human Services, Bethesda, Maryland, USA;; 3 Department of Social and Preventive Medicine, State University of New York, Buffalo, New York, USA;; 4 Epidemiology Branch, National Institute of Environmental Health Sciences, National Institutes of Health, Department of Health and Human Services, Research Triangle Park, North Carolina, USA

**Keywords:** agriculture, cancer, multiple myeloma, occupation, permethrin, pesticide applicator, pesticides, pyrethroid

## Abstract

**Background:**

Permethrin is a synthetic pyrethroid insecticide widely used in agriculture, in public health, and in many U.S. homes and gardens.

**Objective:**

In this study we evaluated the incidence of cancer among pesticide applicators exposed to permethrin in the Agricultural Health Study (AHS).

**Methods:**

A total of 49,093 pesticide applicators were included in this analysis of the AHS, a prospective cohort study of licensed pesticide applicators in Iowa and North Carolina. Detailed information on pesticide exposure and lifestyle factors was obtained from self-administered questionnaires completed in 1993–1997. Average length of follow-up since applicator enrollment in the cohort was 9.14 years. We used two permethrin exposure metrics: *a*) lifetime days applicators personally mixed or applied permethrin and *b*) intensity-weighted lifetime days (lifetime days weighted by estimated intensity of exposure). We used Poisson regression analysis to estimate relative risks (RRs) and 95% confidence intervals (CIs) for malignancies by tertiles of exposure.

**Results:**

We found no associations between permethrin and all malignant neoplasms combined, or between permethrin and melanoma, non-Hodgkin lymphoma, leukemia, or cancers of the colon, rectum, lung, or prostate. We found elevated and statistically significant risks for multiple myeloma in the highest tertiles of both lifetime exposure-days (RR = 5.72; 95% CI, 2.76–11.87) and intensity-weighted lifetime exposure-days (RR = 5.01; 95% CI, 2.41–10.42), compared with applicators reporting they never used permethrin; these results are based on only 15 exposed cases. These findings were similar across a variety of alternative exposure metrics, exposure categories, and reference groups.

**Conclusions:**

This study found no association with most cancers analyzed. Although the suggested association with multiple myeloma was based on a small number of cases, it warrants further evaluation.

Permethrin, a broad-spectrum synthetic pyrethroid insecticide registered with the U.S. Environmental Protection Agency (EPA), was first synthesized in 1973 and first marketed in 1977 (Swaine and Tandy 1984). Permethrin was first registered and tolerances were established in the United States in 1979 (U.S. EPA 2006). It is widely used agriculturally on cotton, wheat, corn, alfalfa, and other crops, and approximately 2 million pounds of permethrin are applied annually to agricultural, residential, and public health use sites (U.S. EPA 2006), with > 100 million applications made annually in and around U.S. homes (Whitmore et al. 1992). Applications in the public health arena have focused particularly on insect control in buildings and aircrafts, treatment of mosquito nets, and control of human lice (used in lice shampoos and body lotions for scabies) [International Agency for Research on Cancer (IARC) 1991; National Pesticide Telecommunications Network 1997]. In addition, permethrin is part of the U.S. Department of Defense’s Insect Repellent System (U.S. Department of Defense 2002; Young and Evans 1998). It is the insecticide used on battle dress uniforms (BDUs), because it is considered the most effective clothing impregnant available (U.S. Department of Defense 2002). Permethrin is available in dusts, emulsifiable concentrates, smokes, ultra-low-volume concentrates, and wettable-powder formulations. Exposure can occur via inhalation and dermal contact, and at much lower levels from consumption of food containing residues (Agency for Toxic Substances and Disease Registry 2003; Food and Drug Administration 2004). Permethrin, like other pyrethroids, works by quickly paralyzing the nervous system of insects. It kills insects by contact or by ingestion; it also has repellent effects (National Pesticide Telecommunications Network 1997).

Despite the potential for exposure to applicators during permethrin use and possible exposure to a large proportion of the general population from widespread use, information is limited concerning its potential carcinogenicity and mutagenicity. Mammalian and nonmammalian bioassays and toxicology studies have found potential liver carcinogenicity (Hakoi et al. 1992; Price et al. 2007), benign lung tumors in female mice (Ishmael and Lithfield 1988), lymphocyte DNA damage (Gabbianelli et al. 2004), endocrine disruption (Chen et al. 2002; Go et al. 1999; Kakko et al. 2004; Kim et al. 2004, 2005), genotoxicity (Tisch et al. 2002), and inhibition of gap-junctional intercellular communications (Tateno et al. 1993). The U.S. EPA classifies permethrin (CAS no. 52645-53-1) as a “likely to be carcinogenic to humans” (U.S. EPA 2007), based on findings of increased incidence of benign lung tumors in female mice (Ishmael and Lithfield 1988) and liver tumors in rats (Hakoi et al. 1992) and male and female mice (Ishmael and Lithfield 1988). However, the IARC categorizes permethrin in group 3: not classifiable as to carcinogenicity to humans (IARC 1991). Molecular mechanisms that have been proposed for carcinogenicity are a reduction in the activity of an enzyme involved in the breakdown of the amino acid tryptophan, which in turn can lead to buildup of carcinogenic tryptophan breakdown products (el-Toukhy et al. 1989) and inhibition of gap-junctional intercellular communication (Tateno et al. 1993), that is, chemical communication between cells, an important step in carcinogenesis (Leithe et al. 2006).

To date, the Agricultural Health Study (AHS) is the only study to provide information on human cancer from exposure to permethrin and pyrethroid products (Alavanja et al. 2003, 2004; Lee et al. 2007). Because the AHS questionnaire did not explicitly distinguish products, we refer to “permethrin” throughout this article to represent both permethrin and pyrethroid products. Permethrin has been evaluated for cancer risk in case–control analyses of AHS data for cancers of the prostate (Alavanja et al. 2003), lung (Alavanja et al. 2004), colon (Lee et al. 2007), and breast (Engel et al. 2005). A positive association for prostate cancer was found among applicators with a family history of prostate cancer who ever reported applying permethrin to animals [relative risk (RR) = 2.38; 95% confidence interval (CI), 1.34–4.25], but not among applicators without a family history of prostate cancer (Alavanja et al. 2003). No associations were found with permethrin used on crops (Alavanja et al. 2003), and no associations were found between lung cancer or colon cancer and animal or crop permethrin use (Alavanja et al. 2004; Lee et al. 2007). There was no association between permethrin use and risk of breast cancer among farmers’ wives in the AHS (Engel et al. 2005).

For the present analysis, we investigated site-specific cancer incidence and risk among pesticide applicators exposed to permethrin in the AHS cohort to provide additional information on this important agricultural chemical. Given the increased follow-up time for case accrual, in this study we extend previous analyses for prostate, lung, and colon cancers (Alavanja et al. 2003, 2004; Lee et al. 2007) and examine the relationship between permethrin and additional cancer sites.

## Materials and Methods

### Cohort enrollment and follow-up

The AHS is a prospective cohort study composed of 57,311 private and commercial applicators who were licensed to apply restricted-use pesticides in Iowa or North Carolina at the time of enrollment (82.4% of eligible applicators in both states enrolled) (Blair et al. 1992). Recruitment of the cohort occurred between 1993 and 1997 (Alavanja et al. 1996). Cohort members were matched to cancer registry files in Iowa and North Carolina for case identification and to the state death registries and the National Death Index to ascertain vital status. For this analysis, we identified incident cancers between date of enrollment and 31 December 2004 and coded them according to the *International Classification of Diseases for Oncology, 2nd Revision* (ICD-O-2) (World Health Organization 1990). We identified cohort members no longer residing in Iowa or North Carolina (*n* = 978) through current address searches by the Internal Revenue Service (address information only), motor vehicle registration offices, and pesticide license registries of the state agricultural departments and censored them in the year that they left the state. Individuals were followed until the earliest of first primary cancer diagnosis of any type, death, date they left the state, or 31 December 2004. The average time of follow-up from enrollment was 9.14 years. All participants provided informed consent, and the institutional review boards of the National Cancer Institute (Bethesda, MD), Battelle Centers for Public Health Research and Evaluation (Durham, NC) (field station in North Carolina), University of Iowa (field station in Iowa), and Westat, Inc. (Rockville, MD) (coordinating center for the study) approved the protocol.

### Exposure assessment

A self-administered enrollment questionnaire collected comprehensive exposure data on 22 pesticides; ever/never-use information for 28 additional pesticides; information on use of personal protective equipment (PPE), pesticide application methods, pesticide mixing, pesticide equipment repair, smoking, alcohol consumption, and cancer history of first degree relatives; and basic demographic data (Alavanja et al. 1999). Reliability of pesticide reporting has been evaluated, and percentage agreement is in the 70–90% range for ever/never use of individual pesticides and in the 50–60% range for duration, frequency, and decade of use (Blair et al. 2002). The questionnaire may be accessed online (National Institutes of Health 2008).

In the AHS, two types of permethrin exposure were assessed: permethrin used on animals and permethrin or pyrethroid products used on crops. Because these two uses have a similar mix of compounds, for this study we combined data for both permethrin uses, constructed two lifetime permethrin exposure metrics (lifetime exposure-days and intensity-weighted lifetime exposure-days), and categorized each into tertiles. We based the lifetime exposure-days metric on the number of years an applicator personally applied or mixed permethrin multiplied by the number of days in an average year an applicator personally mixed or applied permethrin. We used the midpoints of the questionnaire categories to calculate the product of years of use times days per year. We summed the lifetime exposure-days for animal-use permethrin and for crop-use permethrin to yield our permethrin lifetime days metric (tertiles: < 8.75, 8.75–50.75, > 50.75 days).

The intensity-weighted lifetime exposure-days metric was the product of lifetime exposure-days and intensity level, which incorporates factors that may influence the extent of exposure during pesticide mixing and application. We used enrollment questionnaire data in an algorithm to estimate intensity of exposure to individual pesticides as follows: intensity level = [(mixing status + application method + equipment repair status) × PPE use] (Dosemeci et al. 2002). We assigned the scores to each factor in the intensity-level algorithm not as nominal or ordinal values, but as weighted values to reflect likely intensity of exposure (to pesticides, not to permethrin specifically), as described in the literature (Dosemeci et al. 2002). Mixing status was a three-level variable based on never mixing, personally mixing < 50% of the time, and personally mixing ≥ 50% of the time (mixing status = 0, 3, and 9, respectively). Application method was a six-level variable based on never applying, use of aerial aircraft or distribution of tablets, application in furrow, use of boom on tractor, use of backpack, and use of hand spray (application method = 0, 1, 2, 3, 8, 9, respectively). Equipment repair status was a two-level variable based on not repairing or repairing (equipment repair status = 0, 2, respectively). PPE use was an eight-level variable based on type of PPE used while applying pesticides. We combined lifetime exposure-days with the measure of intensity (a scored value ranging from 0.2 to 20.0) to create intensity-weighted lifetime exposure-days. We summed intensity-weighted lifetime exposure-days for animal-use and crop-use permethrin to yield our permethrin intensity-weighted lifetime exposure-days metric (tertiles: < 59.5, 59.5–220.5, > 220.5 intensity-weighted lifetime exposure-days).

### Statistical analysis

We excluded from this analysis individuals with prevalent cancers (i.e., those diagnosed before enrollment; *n* = 1,084), who did not provide information on permethrin use (*n* = 6,855), or with missing information on age or person-years of follow-up (*n* = 278), leaving 49,093 applicators included in the analyses. Analyses of first primary incident cancer cases enabled us to obtain exposure data from each case before the onset of cancer.

We fit Poisson regression models for individual cancer sites to estimate RRs associated with tertiles of lifetime exposure-days or intensity-weighted exposure-days. We investigated all cancer sites classified under ICD-O-2, but present results only for cancers for which there were at least 15 permethrin-exposed cases, after accounting for missing covariate data. Cancer sites meeting this criterion included all cancers combined; cancers of the colon, rectum, lung, prostate, and bladder; melanoma; all lymphohematopoietic system cancers; leukemia; multiple myeloma; and non-Hodgkin lymphoma. We adjusted rate ratios for age at enrollment (< 40, 40–49, 50–59, ≥ 60), sex, race (white, nonwhite), family history in first-degree relative of the specific cancer being analyzed (yes, no), cigarette smoking (never/low/high: we used the median value of pack-years among smokers to classify low and high categories of smokers), state of residence (Iowa/North Carolina), and enrollment year. Because of potential concomitant exposure to other pesticides, we investigated the correlations between permethrin use and use of other pesticides. We calculated Spearman correlation coefficients (*r*) for lifetime days and for intensity-weighted lifetime exposure-days of permethrin with all other pesticides in the AHS. For lifetime exposure-days, the five pesticides most highly correlated with permethrin were trichlorfon (*r* = 0.35), ziram (*r* = 0.24), coumaphos (*r* = 0.19), chlorothalonil (*r* = 0.19), and aldicarb (*r* = 0.18); for intensity-weighted lifetime exposure-days, the five most highly correlated pesticides were dichlorvos (*r* = 0.25), cyanazine (*r* = 0.24), metolachlor (*r* = 0.22), atrazine (*r* = 0.22), and alachlor (*r* = 0.22) Because these correlation coefficients were small, we considered whether they should be included in the final models by first including them and then removing them from the models for lifetime exposure-days and intensity-weighted lifetime exposure-days. We analyzed exposure–response trends by including the midpoint of each tertile as a continuous variable in the model and testing for the statistical significance of the slope.

To evaluate the more appropriate reference group for RRs—applicators who never applied permethrin (hereafter referred to as “non-permethrin-exposed applicators”) or applicators in the lowest exposure tertile of permethrin, we compared baseline characteristics of each of these groups with those of applicators with permethrin exposure in the highest tertiles (for lifetime exposure-days). We considered applicators with baseline characteristics more similar to those of the applicators in the higher exposure group to be more appropriate as referents for the Poisson regression analyses, because differences with respect to baseline characteristics (Table 1) might introduce residual confounding from a variety of unidentified sources.

We carried out additional analyses on prostate cancer to compare our results with those of the nested case–control analysis of prostate cancer in the AHS (Alavanja et al. 2003). For these analyses, we stratified by family history of prostate cancer, compared applicators ever exposed to permethrin with those never exposed, and calculated an interaction RR.

We conducted all statistical analyses with Stata (version 9.0; StataCorp, College Station, TX). We used the P1REL0612 release of the AHS database (www.aghealth.org).

## Results

Table 1 presents selected characteristics of the permethrin-exposed and non-permethrin-exposed applicators, categorized by lifetime exposure-days [nonexposed, lower exposed (lowest tertile), and higher exposed (top two tertiles combined)]. Among 49,093 subjects with complete exposure information, 11,623 (24%) reported ever having personally applied or mixed permethrin and had complete data on lifetime days of use. The cohort, both exposed and nonexposed, consisted primarily of white, male private applicators. This is a population with relatively low smoking rates; about half of the applicators reported that they had never smoked. Because there were no pronounced or consistent differences across the three groups for baseline characteristics, we determined that either the nonexposed applicators or the applicators in the lowest tertile of exposure would be a reasonable reference group, but they have slightly different assumptions. Although we carried out analyses using both reference groups, we present only RRs based on the nonexposed group in Table 2.

Table 2 presents the Poisson regression RRs of selected cancers with tertiles of exposure to permethrin, for lifetime days and for intensity-weighted lifetime exposure-days. Including the most highly correlated pesticides in the final models did not appreciably change the estimates, so we opted for a more parsimonious model. We observed no significant variation in RRs for all cancers combined, bladder cancer, colon cancer, lung cancer, melanoma, non-Hodgkin lymphoma, prostate cancer, and rectum cancer, although for rectum and lung cancers and melanoma RRs tended to be small among the more heavily exposed applicators. For all lymphohematopoietic cancers combined, there was a statistically significant increased risk in the top tertile of permethrin lifetime exposure-days (RR = 1.64; 95% CI, 1.07–2.52), but the test for trend was not statistically significant (*p* = 0.35). The RR estimate for the top tertile of permethrin intensity-weighted lifetime exposure-days was elevated but not statistically significant for all lymphohematopoietic cancers (RR = 1.31; 95% CI 0.84–2.04; *p*-trend = 0.60). We found a similar pattern for leukemia (a lymphohematopoietic cancer), in that the lifetime exposure-days estimate was elevated but not statistically significant (RR = 1.74; 95% CI, 0.83–3.64; *p*-trend = 0.60) and the RR for the intensity-weighted lifetime exposure-days metric was smaller (RR = 1.34; 95% CI, 0.61–2.92; *p*-trend = 0.95). We found an elevated risk for multiple myeloma among applicators in the highest tertile (*n* = 10) of lifetime exposure-days compared with nonexposed applicators (RR = 5.72; 95% CI, 2.76–11.87); the *p*-trend was statistically significant (< 0.01), but the RRs did not increase monotonically. We also found an elevation of risk for the highest tertile of intensity-weighted lifetime exposure-days metric (RR = 5.01; 95% CI, 2.41–10.42; *p*-trend < 0.01). Compared with the lowest exposed group, we found statistically significant, elevated RRs in the upper tertile of exposure both for lifetime exposure-days (RR = 4.76; 95% CI, 1.29–17.52; *p*-trend = 0.01) and for intensity-weighted lifetime exposure-days (RR = 5.32; 95% CI, 1.15–24.54; *p*-trend = 0.02; data not shown). For all cancers combined and for other individual cancers, results were similar when the referent was the lowest exposed group (data not shown).

Among applicators with a family history of prostate cancer, we found little evidence of an association with use of permethrin when comparing those ever exposed with those never exposed (RR = 1.19; 95% CI, 0.82–1.3). Those without a family history showed a slight deficit (RR = 0.88; 95% CI, 0.72–1.07). The interaction term was statistically significant (RR = 1.61; 95% CI, 1.08–2.40).

We also carried out all our Poisson regression analyses on animal-use permethrin and crop-use permethrin separately (data not shown) for both lifetime exposure-days and intensity-weighted lifetime exposure-days, using the two reference groups; results in both groups were similar to those for the combined permethrin results.

## Discussion

We found no association between permethrin and all cancers combined; cancers of the colon, rectum, lung, prostate, and bladder; melanoma; and non-Hodgkin lymphoma. Some RR estimates for all lymphohematopoietic cancers and for leukemia were elevated (lifetime days), but findings were not consistent across exposure metrics. We found an elevated risk for multiple myeloma among applicators in the highest tertile (*n* = 10) of lifetime exposure-days (RR = 5.72; 95% CI, 2.76–11.87; *p*-trend < 0.01) and in the highest tertile of intensity-weighted lifetime exposure-days (RR = 5.01; 95% CI, 2.41–10.42; *p*-trend < 0.01) compared with nonexposed applicators. We also found consistently elevated and statistically significant risks for multiple myeloma for both exposure metrics and when using applicators in the lowest tertile of exposure as the reference group. However, there were only 15 exposed cases; small numbers indicate this could be a chance finding.

To further evaluate the multiple myeloma association, we carried out a series of additional analyses using different categorizations of lifetime exposure-days and intensity-weighted lifetime exposure-days, as well as other exposure metrics available to us in the AHS: years applied permethrin, average days per year applied permethrin, and intensity score for permethrin. We found consistently elevated RRs in the top exposure categories and highly significant tests for trend (data not shown). An analysis stratifying by state of residence showed statistically significant elevations in the upper tertile for both Iowa and North Carolina (data not shown).

Human data on cancer and permethrin exposure are limited. The only epidemiologic findings available, based on earlier analyses in the AHS cohort, showed an elevated risk for prostate cancer among applicators with a family history of prostate cancer who ever applied permethrin to animals (RR = 2.38; 95% CI, 1.34–4.25), compared with those who never applied it, but no elevation among applicators who had no family history of prostate cancer (Alavanja et al. 2003). Our results for prostate cancer were weaker. The reasons for this difference are not clear. Our cohort analysis consisted of five additional years of cancer incidence data and was based on a combined use of permethrin on animals and on crops, whereas the analysis by Alavanja et al. (2003) was a case–control analysis based on the use of permethrin on animals. However, in an analysis for animal permethrin alone, we found no strong association. Other case–control analyses in the AHS found no evidence of elevated risk for cancer of the lung (Alavanja et al. 2004), colon (Lee et al. 2007), or breast (Engel et al. 2005) with permethrin exposure; we also found no evidence of elevated risk for these cancers in this analysis. Chemical-specific analyses from the AHS have shown non-statistically significant increases in multiple myeloma with glyphosate use (De Roos et al. 2005) and atrazine use (Rusiecki et al. 2004). However, adjustment for atrazine, glyphosate, and other highly correlated pesticides did not affect the results, so the association between multiple myeloma and permethrin observed here is unlikely to be due to confounding by other pesticide exposures.

Before introducing permethrin-impregnated BDUs for military personnel, the U.S. Army asked the National Research Council (NRC) to review the toxicologic and exposure data on permethrin and perform a quantitative risk assessment to evaluate health risks to deployed U.S. military personnel from vector management tactics. The NRC risk assessment was used to determine whether wearing BDUs impregnated with permethrin (at a concentration of 0.125 mg/cm^2^ of fabric) 18 hr/day, 7 days/week, for up to 10 years is safe for soldiers, and whether handling permethrin-impregnated fabric is safe for garment workers. The aggregate cancer risk for permethrin, based on estimated exposures for various scenarios and pathways, was found to be low (Macedo et al. 2007). The NRC concluded that, based on the review of toxicity data on permethrin, soldiers who wear permethrin-impregnated BDUs are unlikely to experience adverse health effects (NRC 1994).

There are no previous human data on a potential link between permethrin exposure and multiple myeloma. Multiple myeloma is an incurable B-cell malignancy morphologically characterized by a proliferation of plasma cells in the bone marrow (Kyle and Rajkumar 2004). It is often preceded by a clinically benign and typically asymptomatic precursor condition, monoclonal gammopathy of undetermined significance (MGUS) (Kyle et al. 2002; Landgren et al. 2006). However, it remains unclear whether MGUS precedes all cases of multiple myeloma, or if multiple myeloma can arise *de novo* without preceding MGUS (Hideshima et al. 2007). To date, there are no established lifestyle, occupational, or environmental risk factors for MGUS and multiple myeloma (Landgren and Kyle 2007). Because the risk of progression from MGUS to multiple myeloma in the general population has been reported to be very stable regardless of the duration of antecedent MGUS (Kyle et al. 2002; Landgren et al. 2006), it has been proposed to reflect the second hit in a random, two-hit genetic model of malignancy (Rajkumar 2005). The specific second hit that initiates the cascade of events associated with progression is unknown but may include gene–environment interactions. Permethrin might play a role as an immune modulatory factor (via immune stimulation, immune dysregulation, or both) involved in multiple myeloma progression. Alternatively, permethrin might act in a similar fashion and trigger the development of MGUS, which in turn is reflected in an excess risk of multiple myeloma, or the observed elevated risk of multiple myeloma could simply be a chance finding.

A hypothesis proposed for the potential carcinogenicity of permethrin involves the breakdown of an amino acid, tryptophan, which can in turn lead to buildup of carcinogenic tryptophan breakdown products (el-Toukhy et al. 1989) and inhibition of gap-junctional intercellular communication (Tateno et al. 1993). Mechanisms of action, however, are often dose dependent, and because the AHS does not include dose information, our discussion of mechanisms and biological plausibility must be limited at this time. In experimental studies, permethrin has been evaluated for carcinogenic activity in both rats and mice and for mutagenic activity *in vitro*. Results of cancer bioassays in laboratory animals are mixed, and none have indicated an increased risk for multiple myeloma or other hematopoietic cancers (el-Toukhy et al. 1989; Gabbianelli et al. 2004; Hakoi et al. 1992; IARC 1991; Ishmael and Lithfield 1988; Tisch et al. 2002).

Certain limitations of our data hinder the inferences we can make regarding cancer risks from permethrin use. Although the AHS cohort is large, and 11,688 participants reported permethrin use, the small numbers of certain cancers occurring during the 9.14-year average follow-up period resulted in relatively imprecise risk estimates. In addition, most permethrin applicators were male (98%), precluding our ability to assess the association between permethrin exposure and female cancers. Another limitation is that almost all applicators identified themselves as white (99%). Our analysis provides limited information on the timing of pesticide use in relation to disease. Additionally, with only 9.14 years of follow-up, we are limited in our conclusions concerning latency and temporal changes in PPE. We will better address these issues with increased follow-up and exposure data from subsequent phases of the AHS. Although our study used more detailed exposure estimates than did earlier studies, the hours per day applicators engaged in pesticide application could vary considerably. Finally, although the exposure scale in this study is more sophisticated than that employed in most epidemiologic studies of pesticides, undoubtedly considerable exposure misclassification still occurs, which would tend to bias risk estimates in a prospective study such as this toward the null.

The AHS has several important strengths. It is the largest study of pesticide applicators exposed to permethrin to date. Exposure information was gathered before cancer diagnosis, thereby minimizing recall bias. In general, farmers provide reliable information and considerable detail regarding their pesticide application history (Blair and Zahm 1993; Blair et al. 1997, 2002; Coble et al. 2002). The AHS cohort consists of licensed pesticide applicators who are responsible for a thorough understanding of pesticide regulations and for the purchase and application of chemicals (Hoppin et al. 2002). Recall of pesticide use by the AHS cohort has been shown to be consistent with the dates these pesticides came onto the market (Hoppin et al. 2002). Comprehensive questionnaire data was used to quantify permethrin exposure levels, providing discrimination between high and low exposures, rather than defining exposure as “ever used” permethrin. In addition, detailed information on the use of many common pesticides and lifestyle characteristics allowed us to adjust for potential confounding factors.

Despite the limitations noted above, our prospective study of cancer incidence among permethrin-exposed pesticide applicators provided an opportunity afforded in few other studies to evaluate cancer risks associated with exposure to this very widely used pesticide, while adjusting for lifestyle factors. We found no evidence of increased risk of cancer for most of the sites we investigated. There was a suggestion of an increased risk for multiple myeloma with increased lifetime exposure-days and intensity-weighted lifetime exposure-days and other exposure metrics (average days per year, total years, intensity level). However, the number of exposed multiple myeloma cases was small, and we cannot rule out that these findings may have occurred by chance. We intend to follow up these results in the future, focusing specifically on multiple myeloma as more cases develop in the cohort.

## Figures and Tables

**Table 1:** Characteristics of study participants in permethrin chemical-specific analyses [no. (%)].

Characteristic	Nonexposed (*n* = 37,470)	Lowest exposed tertile lifetime days (*n* = 4,325)	Two highest tertiles lifetime days (*n* = 7,298)
Age (years)			
< 40	12,099 (32.3)	1,593 (36.8)	2,853 (39.1)
40–49	10,179 (27.2)	1,418 (32.79)	2,506 (34.3)
50–59	7,852 (21.0)	847 (19.6)	1,262 (17.3)
≥ 60	7,340 (19.6)	467 (10.8)	677 (9.3)

Sex			
Males	36,399 (97.1)	4,257 (98.4)	7,167 (98.2)
Females	1,071 (2.9)	68 (1.6)	131 (1.8)

Race			
White	36,455 (97.3)	4,276 (98.9)	7,190 (98.5)
Other	929 (2.5)	38 (0.9)	92 (1.3)
Missing	96 (0.3)	11 (0.3)	16 (0.2)

Residence			
Iowa	24,896 (66.4)	3,442 (79.6)	5,000 (68.5)
North Carolina	12,574 (33.6)	883 (20.4)	2,298 (31.5)

Applicator			
Commercial	3,284 (8.8)	297 (6.9)	872 (12.0)
Private	34,186 (91.2)	4,028 (93.13)	6,426 (88.1)

Smoking (pack-years)			
0	19,613 (52.3)	2,513 (58.1)	4,082 (55.9)
< 12	8,227 (22.0)	947 (21.9)	1,579 (21.6)
12–195	8,291 (22.1)	770 (17.8)	1,453 (19.9)
Missing	1,339 (3.6)	95 (2.2)	184 (2.5)

Alcohol frequency per year (no. of drinks)			
0	12,217 (32.6)	1,008 (23.3)	1,771 (24.3)
≤ 30	24,721 (66.0)	3,278 (75.8)	5,433 (74.5)
Missing	532 (1.4)	39 (0.9)	94 (1.3)

Education			
≤ High school equivalent	21,437 (57.2)	1,951 (45.1)	3,237 (44.4)
> High school	15,242 (40.7)	2,316 (53.6)	3,920 (53.7)
Missing	791 (2.1)	58 (1.3)	141 (1.9)

Cancer history (first-degree relative)			
No	21,372 (57.0)	2,390 (55.3)	3,968 (54.4)
Yes	13,848 (36.96)	1,763 (40.8)	2,975 (40.8)
Missing	2,250 (6.0)	172 (4.0)	355 (4.9)

Trichlorfon use			
Ever	295 (0.8)	35 (0.8)	168 (2.3)
Never	36,928 (98.6)	4,154 (96.1)	6,831 (93.6)
Missing	247 (0.7)	136 (3.1)	299 (4.1)

Ziram use			
Ever	49 (0.1)	16 (0.4)	42 (0.6)
Never	16,286 (43.5)	1,956 (45.2)	3,013 (41.3)
Missing	21,135 (56.4)	2,353 (54.4)	4,243 (58.1)

Coumaphos use			
Ever	2,053 (5.5)	541 (12.5)	1,140 (15.6)
Never	34,918 (93.2)	3,605 (83.4)	5,831 (79.9)
Missing	499 (1.3)	179 (4.1)	327 (4.5)

Chlorothalonil use			
Ever	2,413 (6.4)	405 (9.36)	1,307 (17.9)
Never	34,935 (93.2)	3,885 (89.8)	5,935 (81.3)
Missing	122 (0.3)	35 (0.8)	56 (0.8)

Aldicarb use			
Ever	1,017 (2.7)	158 (3.7)	448 (6.1)
Never	15,257 (40.7)	1,808 (41.8)	2,621 (35.9)
Missing	21,196 (56.6)	2,359 (54.5)	4,229 (58.0)

**Table 2:** Rate ratios and 95% CIs^a^ for selected cancers with permethrin exposure, comparing each tertile of exposure with nonexposed applicators.

		Lifetime exposure-days			Intensity-weighted lifetime exposure-days		
Cancer (ICD-O-2 code)	Tertile^b^	No.	RR (95% CI)	*p*-Trend	No.	RR (95% CI)	*p*-Trend
All malignant neoplasms (codes 140–208)	0	2,059	1.00 (referent)		2,059	1.00 (referent)	
	1	171	0.92 (0.79–1.08)		141	0.83 (0.70–0.99)	
	2	128	0.84 (0.70–1.00)		142	0.98 (0.82–1.16)	
	3	129	0.94 (0.79–1.12)	0.09	142	0.90 (0.75–1.06)	0.12
Colon (code 153)	0	155	1.00 (referent)		155	1.00 (referent)	
	1	13	0.96 (0.54–1.69)		11	0.89 (0.48–1.64)	
	2	7	0.63 (0.30–1.35)		10	0.94 (0.49–1.78)	
	3	13	1.32 (0.75–2.34)	0.91	12	1.07 (0.59–1.93)	1.00
Rectum (code 154)	0	87	1.00 (referent)		87	1.00 (referent)	
	1	7	0.85 (0.39–1.85)		6	0.81 (0.35–1.86)	
	2	4	0.58 (0.21–1.59)		5	0.77 (0.31–1.90)	
	3	5	0.78 (0.31–1.92)	0.29	5	0.67 (0.27–1.65)	0.28
Lung (code 162)	0	204	1.00 (referent)		204	1.00 (referent)	
	1	13	0.85 (0.49–1.50)		14	1.01 (0.59–1.74)	
	2	8	0.60 (0.30–1.22)		7	0.58 (0.27–1.24)	
	3	9	0.69 (0.35–1.34)	0.09	9	0.57 (0.29–1.12)	0.05
Melanoma (code 172)	0	84	1.00 (referent)		84	1.00 (referent)	
	1	9	1.01 (0.50–2.01)		7	0.85 (0.39–1.85)	
	2	6	0.79 (0.35–1.83)		6	0.84 (0.37–1.93)	
	3	0	—	0.02	2	0.25 (0.06–1.01)	0.05
Prostate (code 185)	0	853	1.00 (referent)		853	1.00 (referent)	
	1	82	1.11 (0.89–1.40)		60	0.89 (0.68–1.16)	
	2	53	0.89 (0.68–1.18)		69	1.20 (0.94–1.53)	
	3	44	0.87 (0.64–1.18)	0.37	50	0.87 (0.65–1.16)	0.83
Bladder (code 188)	0	97	1.00 (referent)		97	1.00 (referent)	
	1	6	0.74 (0.32–1.70)		6	0.83 (0.36–1.89)	
	2	3	0.46 (0.15–1.46)		4	0.64 (0.24–1.75)	
	3	8	1.33 (0.64–2.74)	0.82	7	1.00 (0.46–2.15)	0.61
All lymphohematopoietic cancers (codes 200–208)	0	207	1.00 (referent)		207	1.00 (referent)	
	1	17	0.85 (0.51–1.39)		15	0.83 (0.49–1.40)	
	2	10	0.61 (0.32–1.16)		13	0.83 (0.47–1.46)	
	3	24	1.64 (1.07–2.52)	0.35	22	1.31 (0.84–2.04)	0.60
Non-Hodgkin lymphoma (codes 200–202)	0	94	1.00 (referent)		94	1.00 (referent)	
	1	8	0.84 (0.41–1.74)		7	0.81 (0.38–1.76)	
	2	5	0.64 (0.26–1.68)		7	0.94 (0.43–2.02)	
	3	5	0.69 (0.28–1.71)	0.22	4	0.48 (0.18–1.31)	0.18
Multiple myeloma (code 203)	0	29	1.00 (referent)		29	1.00 (referent)	
	1	3	1.21 (0.37–3.99)		2	0.92 (0.22–3.85)	
	2	2	1.02 (0.24–4.31)		3	1.55 (0.47–5.12)	
	3	10	5.72 (2.76–11.87)	< 0.01	10	5.01 (2.41–10.42)	< 0.01
Leukemia (code 204–208)	0	72	1.00 (referent)		72	1.00 (referent)	
	1	5	0.75 (0.30–1.87)		5	0.83 (0.33–2.06)	
	2	3	0.56 (0.18–1.78)		3	0.58 (0.18–1.86)	
	3	8	1.74 (0.83–3.64)	0.60	7	1.34 (0.61–2.92)	0.95

a Adjusted for age, sex, race, family history of cancer, cigarette smoking, state of residence, and enrollment year.

b Lifetime days tertiles: 0, never exposed; 1, ≤ 8.75; 2, 8.74–50.75; 3 > 50.75; intensity-weighted lifetime days tertiles: 0, never exposed; 1, < 59.5; 2, 59.5–220.5; 3 ≥ 220.5.
